# HCHS-Net: A Multimodal Handcrafted Feature and Metadata Framework for Interpretable Skin Lesion Classification

**DOI:** 10.3390/biomimetics11020154

**Published:** 2026-02-19

**Authors:** Ahmet Solak

**Affiliations:** Department of Electrical and Electronics Engineering, Faculty of Engineering and Natural Sciences, Konya Technical University, Konya 42250, Türkiye; asolak@ktun.edu.tr

**Keywords:** clinical metadata, computer-aided diagnosis, ensemble gradient boosting, handcrafted features, interpretable machine learning, skin lesion classification

## Abstract

Accurate and timely classification of skin lesions is critical for early cancer detection, yet current deep learning approaches suffer from high computational costs, limited interpretability, and poor transparency for clinical deployment. This study presents HCHS-Net, a lightweight and interpretable multimodal framework for six-class skin lesion classification on the PAD-UFES-20 dataset. The proposed framework extracts a 116-dimensional visual feature vector through three complementary handcrafted modules: a Color Module employing multi-channel histogram analysis to capture chromatic diagnostic patterns, a Haralick Module deriving texture descriptors from the gray-level co-occurrence matrix (GLCM) that quantify surface characteristics correlated with malignancy, and a Shape Module encoding morphological properties via Hu moment invariants aligned with the clinical ABCD rule. The architectural design of HCHS-Net adopts a biomimetic approach by emulating the hierarchical information processing of the human visual system and the cognitive diagnostic workflows of expert dermatologists. Unlike conventional black-box deep learning models, this framework employs parallel processing branches that simulate the selective attention mechanisms of the human eye by focusing on biologically significant visual cues such as chromatic variance, textural entropy, and morphological asymmetry. These visual features are concatenated with a 12-dimensional clinical metadata vector encompassing patient demographics and lesion characteristics, yielding a compact 128-dimensional multimodal representation. Classification is performed through an ensemble of three gradient boosting algorithms (XGBoost, LightGBM, CatBoost) with majority voting. HCHS-Net achieves 97.76% classification accuracy with only 0.25 M parameters, outperforming deep learning baselines, including VGG-16 (94.60%), ResNet-50 (94.80%), and EfficientNet-B2 (95.16%), which require 60–97× more parameters. The framework delivers an inference time of 0.11 ms per image, enabling real-time classification on standard CPUs without GPU acceleration. Ablation analysis confirms the complementary contribution of each feature module, with metadata integration providing a 2.53% accuracy gain. The model achieves perfect melanoma and nevus recall (100%) with 99.55% specificity, maintaining reliable discrimination at safety-critical diagnostic boundaries. Comprehensive benchmarking against 13 published methods demonstrates that domain-informed handcrafted features combined with clinical metadata can match or exceed deep learning fusion approaches while offering superior interpretability and computational efficiency for point-of-care deployment.

## 1. Introduction

Skin cancer is one of the most prevalent malignancies worldwide, with incidence rates increasing steadily over the past decades due to prolonged ultraviolet radiation exposure, changing lifestyles, and an aging global population [1]. Among skin cancer subtypes, melanoma accounts for the highest mortality rate despite representing a relatively small proportion of cases, while non-melanoma skin cancers, such as basal cell carcinoma and squamous cell carcinoma, impose a substantial burden on healthcare systems through their high prevalence [2]. Early and accurate diagnosis remains the cornerstone of effective treatment, as five-year survival rates for melanoma exceed 99% when detected at localized stages but drop below 35% in metastatic cases [3]. However, clinical diagnosis through visual inspection and dermoscopy is inherently subjective, with diagnostic accuracy among dermatologists ranging between 49% and 81% depending on experience level and lesion complexity [4]. This diagnostic variability, coupled with the global shortage of dermatology specialists, particularly in low- and middle-income countries, underscores the urgent need for automated computer-aided diagnostic (CAD) systems that can provide consistent, objective, and accessible skin lesion classification [5].

Advances in deep learning have significantly transformed medical image analysis, with convolutional neural networks (CNNs) achieving dermatologist-level performance in several binary classification studies [6]. Architectures such as ResNet, DenseNet, VGG, and EfficientNet, pre-trained on large-scale datasets through transfer learning, have become the dominant paradigm for skin lesion classification [7,8]. More recently, feature fusion strategies that concatenate representations from multiple CNN backbones have further improved accuracy by capturing complementary visual patterns, including edges, textures, and color distributions [9]. Despite these achievements, deep learning approaches face several persistent challenges that limit their clinical adoption. The high computational cost associated with multi-million parameter models restricts deployment on resource-constrained devices such as smartphones and point-of-care systems [10]. The “black-box” nature of learned feature representations hinders clinical trust and regulatory compliance, as clinicians cannot directly interpret which visual characteristics drive individual predictions [11]. Furthermore, deep models are susceptible to overfitting on small or imbalanced medical datasets and may fail to generalize across imaging modalities, particularly between dermoscopic and smartphone-captured clinical images [12].

Handcrafted feature extraction offers a compelling alternative that addresses many of these limitations. Classical descriptors such as color histograms, texture features derived from the gray-level co-occurrence matrix (GLCM), and shape moments have a long-standing history in medical image analysis, providing domain-specific representations with clear clinical interpretability [13]. Color-based features capture the chromatic heterogeneity that distinguishes malignant from benign lesions, texture descriptors quantify surface patterns correlated with histological characteristics of malignancy, and shape invariants encode the asymmetry and border irregularity formalized in the clinical ABCD rule for melanoma detection [14,15]. Unlike deep features, these descriptors are computationally lightweight, require no GPU acceleration, and produce fixed-dimensional representations that can be directly examined by clinicians [16]. Recent studies have demonstrated that carefully engineered handcrafted pipelines combined with classical machine learning classifiers can achieve competitive performance against deep learning models, particularly when domain knowledge is effectively encoded into the feature design [17,18].

Beyond visual features, clinical metadata such as patient age, gender, lesion location, and symptomatology provide valuable diagnostic context that dermatologists routinely consider during examination [19]. The PAD-UFES-20 dataset, a publicly available benchmark comprising smartphone-captured clinical images with comprehensive patient metadata across six diagnostic categories, presents a particularly relevant testbed for multimodal classification systems [20]. While several studies have attempted metadata integration through attention-based mechanisms and sentence embeddings, these approaches have yielded modest improvements over image-only baselines, suggesting that the effectiveness of metadata fusion depends critically on the discriminative quality of the accompanying visual representations [21,22]. This observation motivates the development of a framework that combines high-quality handcrafted visual features with clinical metadata in a principled multimodal architecture.

The challenge of automated skin lesion classification lies in digitizing the complex visual discrimination capabilities that human experts develop through biological evolution and extensive clinical experience. In this context, the proposed HCHS-Net is built upon a biomimetic feature extraction strategy that draws inspiration from the human visual system’s ability to filter noise and focus on critical diagnostic markers. By encoding morphological and chromatic indicators—such as asymmetry and color variegation—into structured mathematical descriptors, the framework bridges the gap between artificial decision-making and biological perception. Furthermore, the architecture implements a multimodal fusion strategy that mimics how the human brain synthesizes disparate information streams, such as combining visual texture with contextual patient history to form a diagnosis. This biomimetic synergy allows the system to achieve high diagnostic accuracy with minimal computational overhead, echoing the resource-efficient information processing found in biological organisms and offering a more transparent alternative to “black-box” deep learning models.

This study proposes HCHS-Net (Handcrafted Color-Haralick-Shape Network), a lightweight and interpretable multimodal framework for six-class skin lesion classification on the PAD-UFES-20 dataset. The framework extracts a 116-dimensional visual feature vector through three complementary modules: a Color Module based on multi-channel histogram analysis, a Haralick Module capturing texture characteristics via GLCM-derived descriptors, and a Shape Module encoding morphological properties through Hu moment invariants. These visual features are fused with a 12-dimensional clinical metadata vector and classified using an ensemble of three gradient boosting algorithms (XGBoost, LightGBM, CatBoost) with majority voting.

The principal contributions of this work are as follows:

- The primary motivation is to bridge the critical gap between computationally expensive “black-box” deep learning models and the clinical necessity for lightweight, interpretable diagnostic tools suitable for resource-constrained mobile health applications.
- A multimodal handcrafted feature extraction pipeline that directly encodes dermatological diagnostic criteria into a compact 128-dimensional representation.
- A systematic ablation study demonstrating the complementary contribution of each feature module and metadata integration.
- Competitive classification accuracy (97.76%) against state-of-the-art deep learning methods while requiring 60–97× fewer parameters and achieving 28–63× faster inference.
- Comprehensive benchmarking against 13 published methods on the PAD-UFES-20 dataset, establishing that handcrafted features with clinical metadata can match or exceed deep learning fusion approaches in clinically relevant classification tasks.

The remainder of this paper is organized as follows. Section 2 reviews related work on skin lesion classification with emphasis on handcrafted features, deep learning, and multimodal approaches. Section 3 details the proposed HCHS-Net framework, including dataset description, feature extraction modules, metadata integration, and ensemble classification. Section 4 presents experimental results with ablation studies and comparative analysis. Section 5 discusses the findings in the context of existing literature. Section 6 concludes the paper with a summary of contributions and directions for future research.

## 2. Related Works

Research on automated skin lesion classification has evolved from individual deep learning architectures toward increasingly sophisticated methodologies encompassing explainability, computational efficiency, feature fusion, and multimodal integration. Early efforts concentrated on developing high-performing convolutional neural networks (CNNs), though these models were frequently criticized for their “black-box” nature and substantial computational demands. This evolution reflects the field’s growing recognition that clinical deployment requires not only accuracy but also transparency, efficiency, and robustness across diverse imaging conditions.

The quest for specialized architectures optimized for dermatological image analysis has yielded notable advances. Azeem et al. [23] developed SkinLesNet, a multi-layered deep CNN architecture specifically tailored for melanoma detection that achieved 96% accuracy on a three-class subset (melanoma, keratosis, nevus) of the PAD-UFES-20 dataset, surpassing standard transfer learning models such as VGG-19 and ResNet-50. While this customized approach demonstrated superior performance, the inherent opacity of deep networks remained a critical barrier to clinical acceptance. Addressing this limitation, Ashwath et al. [24] proposed TS-CNN, a three-tier self-interpretable architecture designed to enhance decision transparency through feature map visualization, achieving 72.83% accuracy on the full six-class PAD-UFES-20 dataset. This model represents an important step toward explainable AI in dermatology, though its relatively modest accuracy and questions about generalizability across diverse clinical datasets and imaging modalities remain concerns.

Parallel to efforts focused on interpretability, researchers have tackled the computational burden of deep networks, recognizing that resource-constrained deployment scenarios—particularly mobile health applications—demand efficient models. Medhat et al. [25] applied Iterative Magnitude Pruning (IMP) to AlexNet, demonstrating that substantial parameter reduction could be achieved while maintaining an impressive 99.15% accuracy on a binary classification task (melanoma vs. nevus) using PAD-UFES-20. Their findings suggest that deep networks contain considerable redundancy, and strategic pruning can enhance efficiency without compromising performance. This work illuminates a critical trade-off: while deeper, more complex models may capture subtle patterns, they often incorporate unnecessary parameters that inflate computational costs without proportional gains in accuracy.

Feature fusion strategies have emerged as a dominant paradigm for enhancing representational capacity by leveraging complementary strengths of multiple architectures. Bhargavi and Balakrishna [9] introduced DRMv2Net, which concatenates deep features from DenseNet201, ResNet101, and MobileNetV2, achieving 96.12% accuracy on the full six-class PAD-UFES-20 dataset and 96.11% on ISIC-2357. By integrating features across different architectural depths and design philosophies, this approach captures diverse visual patterns—ranging from low-level edges to high-level semantic structures—that individual models might overlook. Building on similar principles, Sasithradevi et al. [26] developed EffiCAT, which augments EfficientNet backbones with Convolutional Block Attention Modules (CBAM). Tested on a four-class subset (ACK, BCC, MEL, NEV) combining HAM10000 and PAD-UFES-20, the model achieved 94.4% accuracy, underscoring the value of attention mechanisms in guiding feature selection toward diagnostically relevant regions. Liu et al. [27] further refined fusion methodologies by proposing a multi-level framework that integrates low-level texture information with high-level semantic representations. While these fusion-based approaches consistently outperform single-model baselines, they introduce increased computational complexity and demand substantial hardware resources, potentially limiting deployment in resource-constrained settings.

An alternative research trajectory combines deep learning feature extraction with classical machine learning classifiers, capitalizing on the interpretability and efficiency of traditional algorithms. Javed et al. [28] employed ConvMixer for deep feature extraction while utilizing Gradient Boosting algorithms—specifically CatBoost, XGBoost, and LightGBM—for classification on the six-class PAD-UFES-20 dataset. Their results revealed that CatBoost demonstrated balanced performance compared to end-to-end deep learning models. This hybrid strategy suggests that shallow classifiers, when paired with robust deep features, can match or exceed purely neural approaches while offering computational advantages. Sathvika et al. [29] reported even more striking results, with a Support Vector Machine (SVM) pipeline fed by dual-partitioned texture features achieving 98.10% accuracy on a five-class subset (excluding SCC)—outperforming the deep learning-based AlexNet model (96.87%). These findings challenge the assumption that deeper is invariably better, indicating that carefully engineered features processed by classical algorithms can rival sophisticated neural architectures. Complementing this trend, Moldovanu et al. [30] emphasized geometric feature extraction through an enhanced iSLIC superpixel algorithm for lesion boundary detection, achieving 98.6% accuracy on a binary classification task (melanoma vs. nevus) with machine learning classifiers. Their work reaffirms the diagnostic value of morphological analysis in skin cancer classification. De et al. [31] extended hybridization to data modalities, developing a CNN-DenseNet model that processes both histopathological and dermatoscopic images, attaining 91.07% accuracy on the full six-class PAD-UFES-20 dataset.

When image data alone proves insufficient for reliable diagnosis, multimodal approaches incorporating patient metadata have shown considerable promise, particularly for challenging lesion differentiation. Pacheco and Krohling [21] pioneered the integration of clinical metadata with image features through MetaBlock, an attention-based fusion mechanism that achieved 76.5% balanced accuracy on the six-class PAD-UFES-20 dataset, outperforming standard concatenation techniques. Although MetaBlock demonstrated the potential of multimodal learning, its performance degraded substantially when metadata was incomplete—a common scenario in real-world clinical practice. Recognizing this critical limitation, Bouzon et al. [32] developed MetaBlock-SE, which leverages sentence embeddings to semantically impute missing metadata. By completing absent clinical information through learned semantic representations, this approach enhanced balanced accuracy to 70.2 ± 2.8% on the six-class PAD-UFES-20 dataset, significantly improving model robustness under missing data conditions. Liu et al. [33] approached multimodal learning from an optimization perspective, proposing a Kernel-based Extreme Learning Machine (KELM) reinforced with Anti-Coronavirus Optimization (ACO) algorithm, achieving 97.9% accuracy on the six-class dataset, emphasizing that intelligent feature selection plays a pivotal role in multimodal system performance.

Despite these advances, the literature reveals persistent challenges that warrant continued investigation, as summarized in Table 1. Class imbalance remains problematic across most benchmark datasets, often requiring extensive data augmentation strategies that may not fully replicate clinical variability. Computational costs associated with deep fusion architectures and ensemble methods pose barriers to deployment in mobile or point-of-care settings, particularly in resource-limited regions where automated diagnostics could have the greatest impact. Generalization across datasets with differing imaging protocols, patient demographics, and lesion distributions continues to challenge model robustness. Moreover, while accuracy rates have approached the 98% threshold in some studies, the clinical utility of these models depends critically on explainability—understanding why a model makes specific predictions—and resilience to variations in image acquisition devices and conditions. Current research has begun addressing these gaps through techniques such as model pruning, attention mechanisms, and metadata integration, yet comprehensive ablation studies examining the interplay between preprocessing strategies, architectural choices, and multimodal fusion remain sparse. Bridging the gap between laboratory performance and clinical deployment necessitates models that balance accuracy, efficiency, interpretability, and adaptability to real-world variability.

## 3. Materials and Methods

This section presents the materials and methods employed in this study. First, the PAD-UFES-20 dataset used for evaluation is described. Then, the proposed HCHS-Net (Handcrafted Color-Haralick-Shape Network) framework is presented in detail. Finally, the baseline methods used for comparative evaluation are introduced.

### 3.1. Dataset Description

The proposed HCHS-Net framework was evaluated on the PAD-UFES-20 dataset, a publicly available clinical skin lesion dataset collected at the Federal University of Espírito Santo (UFES), Brazil [20]. Unlike dermoscopic datasets requiring specialized equipment, PAD-UFES-20 comprises smartphone-captured images under uncontrolled clinical conditions, reflecting real-world diagnostic scenarios in primary healthcare settings.

**Figure 1:**
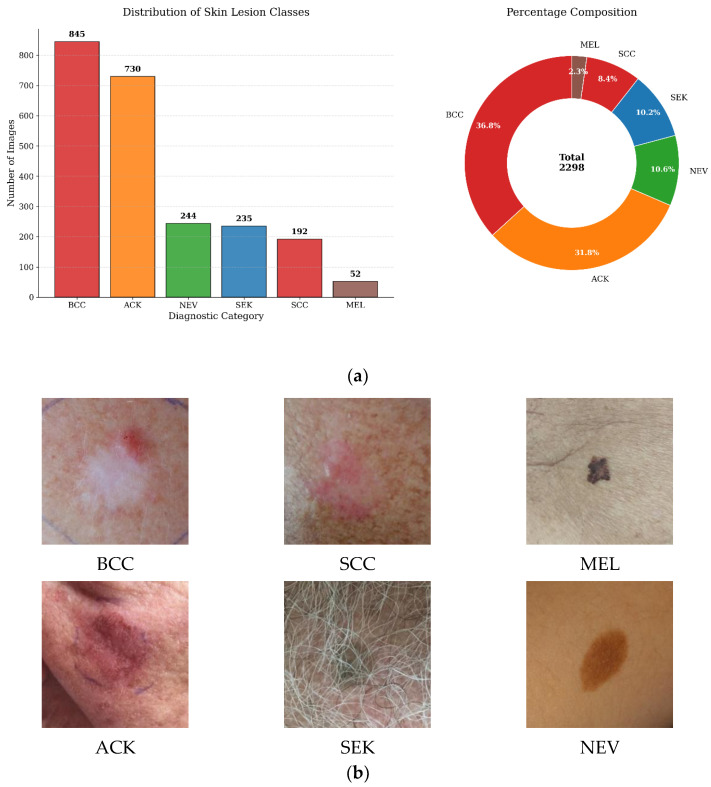
PAD-UFES-20 dataset overview. (**a**) Class distribution showing severe imbalance. (**b**) Representative samples from each class.

The dataset contains 2298 clinical images representing six diagnostic categories of pigmented skin lesions. As shown in Figure 1a and detailed in Table 2, the dataset exhibits severe class imbalance, with melanoma (MEL) containing only 52 samples (2.26%) while basal cell carcinoma (BCC) represents the majority class with 845 samples (36.77%). This imbalance reflects the natural distribution of skin conditions in clinical practice and poses a significant challenge for machine learning models.

Images were captured using smartphone cameras during clinical examinations, exhibiting variable quality, illumination, and artifacts. Representative samples from each diagnostic category are shown in Figure 1b. Original resolutions range from 640 × 480 to 4000 × 3000 pixels, necessitating spatial normalization as part of the preprocessing pipeline.

A distinguishing feature of the PAD-UFES-20 dataset is the inclusion of comprehensive clinical metadata for each patient, as detailed in Table 3. The metadata encompasses demographic information (age, gender), lifestyle factors (smoking, alcohol consumption), lesion dimensions, and symptom presentations (itching, growth, pain, appearance changes, bleeding, elevation). This multimodal information enables the development of classification approaches that integrate visual features with patient-level clinical indicators.

### 3.2. HCHS-Net Framework

HCHS-Net (Handcrafted Color-Haralick-Shape Network) framework is a novel hybrid approach for multi-class skin lesion classification. Unlike deep learning methods that require millions of trainable parameters and extensive computational resources, HCHS-Net leverages domain-specific handcrafted features that capture the visual patterns routinely assessed by dermatologists during clinical examination. The framework comprises five interconnected processing stages: (1) image preprocessing including Gaussian filtering for noise reduction and spatial normalization, (2) offline data augmentation to address severe class imbalance, (3) multimodal handcrafted feature extraction spanning color distribution, texture patterns, and shape characteristics, (4) clinical metadata integration to augment visual features with patient-level diagnostic indicators, and (5) ensemble classification using gradient boosting algorithms that combine predictions from CatBoost, XGBoost, and LightGBM.

**Figure 2:**
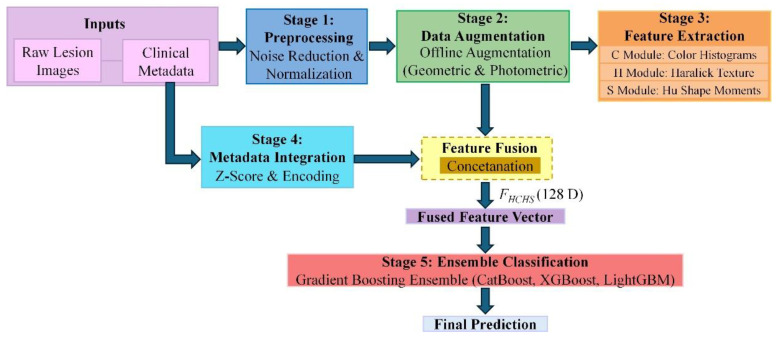
The overall architecture of the proposed HCHS-Net framework, illustrating the five main stages from raw input data to final multi-class classification pipeline.

The naming convention of HCHS-Net reflects its core feature extraction philosophy: handcrafted features derived from color histograms, Haralick texture descriptors, and shape-based Hu moment invariants. This multimodal representation captures complementary visual information that aligns with established dermatological diagnostic criteria such as the ABCD rule for melanoma detection. The complete feature representation comprises only 128 dimensions, offering a dramatic reduction in complexity compared to deep learning models while maintaining superior classification performance. The overall architecture of HCHS-Net is illustrated in Figure 2.

### 3.3. Pre-Processing

This section details the preprocessing pipeline applied to clinical skin lesion images captured from smartphones, utilizing Gaussian filtering for noise reduction and spatial normalization for consistent feature extraction. The preprocessing stage is crucial for handling the inherent variability in smartphone-captured images, which exhibit significant differences in illumination, contrast, and image quality due to diverse acquisition conditions.

The initial step involves applying Gaussian filtering to suppress high-frequency noise and minor artifacts while preserving the diagnostically relevant edge structures and texture patterns essential for accurate lesion classification. A two-dimensional Gaussian kernel with standard deviation *σ = 1.0* is employed, as defined in Equation (1):

(1) $$G\left(x,y\right)=\frac{1}{2\pi \sigma^{2}}\exp\left(-\frac{x^{2}+y^{2}}{2\sigma^{2}}\right)$$

where G (x, y) represents the Gaussian kernel value at position (x, y). As shown in Equation (2), the filtered image *I’* is obtained by convolving the original image *I* with the Gaussian kernel:

(2) $$I^{\prime }\left(x,y\right)=I\left(x,y\right)\times G\left(x,y\right)\;\;\;\;\;\;$$

This filtering operation effectively smooths the image while maintaining the structural integrity of lesion boundaries, which is critical for subsequent shape-based feature extraction. The 5 × 5 kernel size was selected based on empirical evaluation to balance noise suppression with detail preservation.

**Figure 3:**
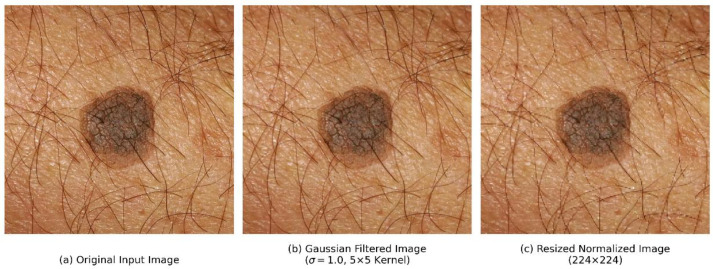
The preprocessing pipeline showing visual transformations: (**a**) example of an original smartphone-captured input image with inherent noise and variable dimensions, (**b**) the image after applying a 5 × 5 Gaussian filter (σ = 1.0) for noise suppression while preserving edges, and (**c**) the final spatially normalized image resized to a standardized 224 × 224 resolution.

Following noise reduction, all images undergo spatial normalization through bilinear interpolation to achieve a standardized resolution of 224 × 224 pixels. This dimension was selected to balance the competing requirements of preserving fine-grained lesion details with computational efficiency considerations. The resizing operation is formulated in Equation (3):

(3) $$I_{224\times 224}=Resize(I^{\prime },(W_{224},H_{224}))\;\;\;\;\;\;$$

where *I’* represents the filtered image and *I_224×224_* represents the resized image with dimensions 224 × 224 pixels. The complete preprocessing pipeline is illustrated in Figure 3, showing (a) original input image, (b) Gaussian filtered image, and (c) resized normalized image.

### 3.4. Data Augmentation

Data augmentation and normalization are essential techniques to improve model robustness and generalization capability, particularly in medical imaging applications where dataset sizes are typically limited [34,35]. As shown in Table 2, the PAD-UFES-20 dataset exhibits severe class imbalance, with melanoma (MEL) containing only 52 samples compared to 845 samples for basal cell carcinoma (BCC). An offline augmentation strategy was therefore implemented to balance the class distribution.

Recent studies have demonstrated that geometric and photometric transformations effectively simulate real-world variability in lesion appearance, improving classification accuracy across different lesion types and orientations [36]. By incorporating such augmentation techniques, models learn to identify skin lesions regardless of positioning or slight morphological differences, thus enhancing their diagnostic reliability. As expressed in Equation (4), the augmented image is obtained through:

(4) $$I_{aug}=Transform(I_{224\times 224},T)\;\;\;\;\;$$

where *I_aug_* represents the augmented image obtained from the resized image by applying transformation set *T*. The complete set of augmentation transformations applied in this study is presented in Table 4.

Through this augmentation process, each of the six diagnostic classes was expanded to contain 2500 samples, yielding a balanced dataset of 15,000 images. Rigorous prevention of data leakage is essential for robust performance estimation. Therefore, the dataset was first partitioned into training (80%) and test (20%) sets at the original image level before any transformations were applied. Data augmentation was subsequently applied exclusively to the training partition to balance the classes. Consequently, the test set contains only genuine, unaugmented images. This rigorous separation ensures that the model’s exceptionally high performance—including the 100% recall on minority classes such as Melanoma—reflects true generalization capabilities rather than the memorization of augmented artifacts derived from a limited number of original samples. The class distribution before and after augmentation is presented in Table 5.

### 3.5. Feature Extraction

**Figure 4:**
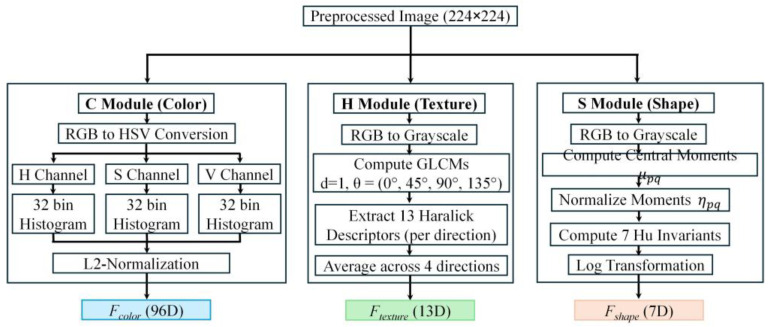
The multimodal handcrafted feature extraction pipeline of HCHS-Net. The preprocessed image is processed in three parallel branches to extract color histograms (HSV space), rotation-invariant Haralick texture features (from GLCM), and shape characteristics (Hu moments), resulting in three distinct feature vectors.

The core innovation of HCHS-Net lies in its multimodal handcrafted feature extraction module, which systematically captures complementary visual information from three distinct domains: color distribution, texture patterns, and shape characteristics. This approach is grounded in clinical dermatology practice, where physicians assess lesions by examining their coloration, surface texture, and morphological properties according to established diagnostic criteria such as the ABCD rule for melanoma detection. The multimodal feature extraction pipeline of HCHS-Net is illustrated in Figure 4.

#### 3.5.1. Color Histogram Features (C-Module)

Color information constitutes a primary diagnostic indicator in dermatological assessment, as different lesion types exhibit characteristic chromatic patterns that reflect underlying histopathological processes. Melanomas typically display multiple colors, including brown, black, red, and blue, while benign nevi tend to exhibit more uniform coloration. HCHS-Net extracts color features in the HSV (Hue-Saturation-Value) color space, which provides a more perceptually meaningful representation than RGB by decoupling chromatic information from intensity.

As shown in Equation (5), the image is first converted from RGB to HSV color space:

(5) $$I_{HSV}=RGB2HSV(I_{224\times 224})\;\;\;\;\;$$

For each color channel *c ∈ (H, S, V)* a 32-bin histogram is computed to capture the global color distribution, as formulated in Equation (6):

(6) $$h_{c}\left(k\right)=\sum_{(x,\;y)\epsilon I}1[b_{k}\leq I_{c}(x,y)<b_{k+1}],\;\;\;\;\;k=0,1,\ldots ,31$$

where b_k_ denotes the boundary of the kth bin and 1[·] is the indicator function. As expressed in Equation (7), the histograms are L2-normalized to achieve invariance to overall image brightness:

(7) $$\hat{h}=\frac{h_{c}}{{\left|h_{c}\right|}_{2}+\varepsilon }$$

where ε is a small constant (10^−8^) for numerical stability. The final color feature vector F_color_ ∈ ℝ^96^ is obtained by concatenating the normalized histograms from all three channels, as shown in Equation (8):

(8) $$F_{color}=\left[{\hat{h}}_{H}\left|{\hat{h}}_{S}\right|{\hat{h}}_{V}\right]$$

#### 3.5.2. Haralick Texture Features (H-Module)

Texture analysis is essential for characterizing the surface patterns of skin lesions, providing crucial information about surface properties that correlate with histological characteristics. Rough, irregular textures often indicate malignancy, while smooth, homogeneous textures are more characteristic of benign lesions. HCHS-Net computes Haralick texture descriptors [13] from the Gray Level Co-occurrence Matrix (GLCM), which encodes the spatial relationship between pixel intensities. As shown in Equation (9), the input image is first converted to grayscale:

(9) $$I_{gray}(x,y)=Grayscale(I_{224\times 224})$$

The GLCM *P_θ,d_(i,j)* represents the probability of observing gray level *j* at distance *d* and angle *θ* from a pixel with gray level *i*, as formulated in Equation (10):

(10) $$P_{\theta ,d}\left(i,j\right)=\;\frac{C_{\theta ,d}\left(i,j\right)}{\sum_{\left(i,j\right)}C_{\theta ,d}\left(i,j\right)}$$

where *C_θ,d_ (i,j)* is the count of co-occurring pixel pairs. GLCMs are computed for four directions θ ∈ (0°, 45°, 90°, 135°) with distance *d = 1* to capture rotation-invariant texture characteristics. From each GLCM, thirteen Haralick descriptors are extracted as detailed in Table 6.

As expressed in Equation (11), the final texture values are obtained by averaging the descriptors across all four directions:

(11) $$F_{texture}=\;\frac{1}{4}\sum_{\theta \;\in \;(0{}^{\circ},\;45{}^{\circ},\;90{}^{\circ},\;135{}^{\circ})\;}H_{\theta }$$

This yields the texture feature vector F_texture_ ∈ ℝ^13^.

#### 3.5.3. Hu Moment Features (S-Module)

Morphological characteristics of skin lesions provide valuable discriminative information, as different lesion types often exhibit distinct contour regularities and shape complexities. Malignant lesions typically display irregular, asymmetric borders, while benign lesions tend to have smooth, symmetric contours. HCHS-Net employs Hu moment invariants [15], which constitute a set of seven statistical descriptors computed from normalized central moments that are mathematically invariant to translation, rotation, and scale transformations.

As shown in Equation (12), the central moments are computed as follows:

(12) $$\mu_{pq}=\;\sum_{x}\sum_{y}{\left(x-\bar{x}\right)}^{p}{\;(y-\bar{y})}^{q}\;I_{gray}(x,y)$$

where $\bar{x}$ and $\bar{y}$ are the centroid coordinates. The normalized central moments are then calculated according to Equation (13):

(13) $$\eta_{pq}=\;\frac{\mu_{pq}}{\mu_{00}^{\frac{p+q}{2}+1}}$$

The seven Hu moments (h_1_, h_2_, …, h_7_) are computed as polynomial combinations of normalized central moments. Given the wide dynamic range of Hu moments spanning several orders of magnitude, a logarithmic transformation is applied to ensure numerical stability during subsequent classification as formulated in Equation (14):

(14) $${\tilde{h}}_{i}\;=\;-sign\left(hi\right)\cdot {log}_{10}\left(\left|h_{i}\right|+\;\varepsilon \right)$$

where ε = 10^−10^ prevents logarithm of zero. This yields the shape feature vector F_shape_ ∈ ℝ^7^.

#### 3.5.4. Feature Fusion

As expressed in Equation (15), the complete HCHS-Net visual feature representation is obtained by concatenating the three complementary feature vectors:

(15) $$F_{visual}=\;\left[F_{color}\right|F_{texture}\left|\;F_{shape}\right]\in \;R^{116}$$

**Figure 5:**
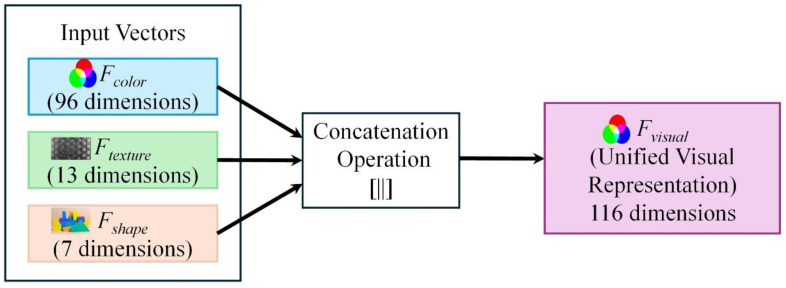
Illustration of the feature fusion process where the complementary feature vectors from color (96D), texture (13D), and shape (7D) modules are concatenated into a unified 116-dimensional visual feature representation (F_visual_).

This multimodal representation captures color distribution (96 dimensions), texture patterns (13 dimensions), and shape characteristics (7 dimensions) in a compact 116-dimensional vector. The feature fusion process, showing how outputs from three extraction modules are merged into a unified representation, is illustrated in Figure 5.

### 3.6. Clinical Metadata Integration

Beyond visual features extracted from lesion images, clinical metadata provides valuable diagnostic information that dermatologists routinely consider during patient examinations. The PAD-UFES-20 dataset includes comprehensive patient metadata that augments the visual representation with clinically relevant indicators not directly observable from lesion images alone. The metadata features are categorized into three types as detailed in Table 7.

As shown in Equation (16), continuous features undergo z-score standardization to achieve zero mean and unit variance:

(16) $$\hat{x}\;=\;(x\;-\;\mu_{x})/(\sigma_{x}\;+\;\varepsilon )$$

where *μ_x_* and *σ_x_* are the mean and standard deviation computed from training data, respectively. As formulated in Equation (17), the metadata feature vector *F_meta_ ∈ ℝ^12^* is concatenated with visual features to form the complete HCHS-Net representation:

(17) $$F\_HCHS\;=\;[F\_visual\;|\;F\_meta]\;=\;[F\_color\;|\;F\_texture\;|\;F\_shape\;|\;F\_meta]\;\in \;R^{128}$$

This compact 128-dimensional feature vector captures multimodal information while maintaining computational efficiency, requiring orders of magnitude fewer parameters than contemporary deep learning models.

### 3.7. Ensemble Classification

**Figure 6:**
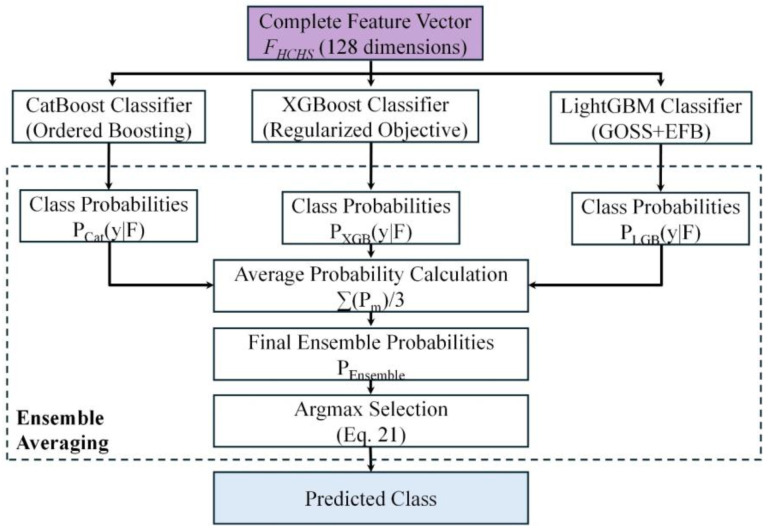
The ensemble classification architecture of HCHS-Net. The complete 128-dimensional feature vector is fed in parallel to three gradient boosting models (CatBoost, XGBoost, LightGBM). The final predictions are obtained by averaging the class probability distributions generated by each individual classifier.

For classification, HCHS-Net employs an ensemble of three state-of-the-art gradient boosting algorithms: CatBoost, XGBoost, and LightGBM. Each classifier offers unique algorithmic advantages for handling tabular data with heterogeneous feature types, and their combination through ensemble averaging provides more robust predictions than individual models. The ensemble classification architecture of HCHS-Net is illustrated in Figure 6.

#### 3.7.1. CatBoost Classifier

CatBoost [37] implements ordered target statistics and ordered boosting to prevent prediction shift, particularly effective for datasets containing categorical features. The model employs symmetric decision trees with oblivious splits, where the same splitting criterion is used across an entire level of the tree. As shown in Equation (18), the prediction for an input feature vector F is computed as follows:

(18) $${\hat{y}}^{t}\;=\sum_{k=1}^{t}\alpha_{k}\;\cdot \;h_{k}(F_{HCHS})$$

where h_k_ represents the kth tree and α_k_ is the corresponding weight.

#### 3.7.2. XGBoost Classifier

XGBoost [38] employs a regularized objective function that explicitly penalizes model complexity to prevent overfitting, as formulated in Equation (19):

(19) $$L\;=\;\sum_{i}\;l(y_{i},\;{\hat{y}}_{i})\;+\;\sum_{k}\;\Omega (hk)$$

where *l* is the loss function and *Ω(h) = γT + ½λ||w||*^2^ penalizes the number of leaves *T* and leaf weights *w*.

#### 3.7.3. LightGBM Classifier

LightGBM [39] utilizes gradient-based one-side sampling (GOSS) and exclusive feature bundling (EFB) for efficient training on large-scale datasets while maintaining prediction accuracy. The leaf-wise growth strategy enables faster convergence compared to level-wise approaches.

#### 3.7.4. Ensemble Strategy

All three classifiers are configured with consistent hyperparameters as presented in Table 8: 500 boosting iterations, maximum tree depth of 6, and learning rate of 0.03.

As expressed in Equation (20), the final classification prediction is obtained by averaging the class probability distributions from all three models:

(20) $$P\left(y=k|F\right)=\frac{1}{3}\sum_{m\in (CAT,\;XGB,LGB)}Pm(y=k|FHCHS)$$

The predicted class is determined according to Equation (21):

(21) $$\hat{y}\;=\;arg\;\underset{k}{\max}\;P(y\;=\;k\;|\;F\_HCHS)$$

This ensemble strategy reduces prediction variance and provides more robust classifications compared to individual models, effectively leveraging the complementary strengths of each gradient boosting algorithm. The complete HCHS-Net pipeline for skin lesion classification is presented in Algorithm 1.
**Algorithm 1** Proposed HCHS-Net FrameworkInput: Raw images X_raw_, Labels Y_raw_, Metadata M_raw_ Output: Predicted Labels Ŷ, Evaluation Metrics 1: PRE-PROCESSING: 2: for each image x ∈ X_raw_ do 3:   Apply Gaussian filtering using Equation (2): x’ = x * G(σ = 1.0) 4:   Resize using Equation (3): x_224_ = Resize (x’, 224 × 224) 5: end for 6: DATA AUGMENTATION (see Table 4): 7: for each class c with count < 2500 do 8:   Apply transformations T using Equation (4) 9:   Generate augmented samples until count = 2500 10: end for 11: FEATURE EXTRACTION: 12: for each preprocessed image x_224_ do 13:   Extract Color features using Equations (6)–(8): F_color_ ∈ ℝ^96^ 14:   Extract Texture features using Equations (10) and (11): Ft_exture_ ∈ ℝ^13^ 15:   Extract Shape features using Equations (12)–(14): F_shape_ ∈ ℝ^7^ 16:   Fuse visual features using Equation (15): F_visual_ = [F_color_ || Ft_exture_ || F_shape_] 17: end for 18: METADATA INTEGRATION: 19: for each metadata vector m ∈ M_raw_ do 20:   Normalize using Equation (16): m_norm_ = Z_Score_(m) 21:   Form F_meta_ ∈ ℝ^12^ (see Table 7) 22: end for 23: FEATURE FUSION using Equation (17): 24: F_HCHS_ = [F_visual_ || F_meta_] ∈ ℝ^128^
25: ENSEMBLE CLASSIFICATION (see Table 8): 26: Train CatBoost, XGBoost, LightGBM using Equations (18) and (19) 27: PREDICTION using Equations (20) and (21): 28: P_ensemble_ = (P_Cat_ + P_XGB_ + P_LGB_)/3 29: Ŷ = argmax (P_ensemble_) 30: return Ŷ, Evaluation Metrics (see Table 9)


### 3.8. Baseline Models and Comparative Evaluation

For comprehensive validation of the effectiveness of HCHS-Net, systematic comparisons were conducted against two categories of baseline methods: classical machine learning approaches and contemporary deep learning architectures. This dual-baseline strategy enables evaluation of HCHS-Net’s performance relative to both traditional feature-based methods and parameter-intensive neural networks.

#### 3.8.1. Classical Machine Learning Baselines

Three established machine learning algorithms were implemented using the identical handcrafted visual features (color histograms, Haralick texture descriptors, and Hu moment invariants) extracted by HCHS-Net. This experimental design isolates the contribution of the classification algorithm from the feature extraction methodology, enabling direct assessment of ensemble boosting effectiveness.

A distance-weighted K-Nearest Neighbors (KNN) classifier with k = 5 neighbors was employed using Euclidean distance metric in the 116-dimensional visual feature space. The value k = 5 was selected through cross-validation on the training set to balance the bias-variance tradeoff. Features were standardized to zero mean and unit variance using z-score normalization to prevent distance metric bias toward high-magnitude features. As a non-parametric instance-based method, KNN maintains negligible model parameters (~0.00 M), offering extremely fast training at the cost of slower inference due to distance computation requirements.

Support Vector Machine (SVM) with Radial Basis Function (RBF) kernel was configured to learn non-linear decision boundaries in the feature space. Hyperparameter optimization via grid search on the training set identified C = 10 (regularization parameter controlling margin softness) and γ = ‘scale’ (RBF kernel coefficient, automatically set to 1/(n_features_ × X.var())) as optimal values. The RBF kernel, defined as K(x,x’) = exp(-γ||x-x’||^2^), enables the model to capture complex non-linear relationships while maintaining computational tractability. Feature standardization was applied identically to KNN. The model contains approximately 0.05 M effective parameters (support vectors and dual coefficients).

Random Forest (RF), an ensemble of 200 decision trees was constructed with maximum depth capped at 20 levels to prevent overfitting while maintaining sufficient model capacity. Each tree was trained on bootstrap samples of the training data with √d features (where d = 116) considered at each split point. The Gini impurity criterion guided node splitting decisions. Class weights were balanced inversely proportional to class frequencies to address dataset imbalance. This ensemble comprises approximately 0.20 M parameters distributed across tree structures and provides implicit feature importance rankings through mean decrease in impurity scores.

All classical models were implemented using scikit-learn 1.2.0 with reproducible random seeds (random_state = 42). Training was performed on the augmented balanced dataset (12,000 samples) with 20% (3000 samples) reserved for testing through stratified splitting.

#### 3.8.2. Deep Learning Baselines

Three representative CNN architectures from distinct design paradigms were selected as deep learning baselines. VGG-16 and ResNet-50 represent the widely adopted sequential and residual learning paradigms, respectively. EfficientNet-B2 was deliberately chosen to represent the modern family of highly optimized, parameter-efficient architectures. This specific selection eliminates the need for redundant benchmarking against other lightweight models, such as MobileNet-V3, as EfficientNet-B2 already establishes a rigorous performance benchmark for computationally optimized CNNs. The primary objective of this comparative analysis focuses on evaluating the handcrafted multimodal pipeline against these established deep learning paradigms, rather than conducting an exhaustive survey of all available mobile architectures. All models were initialized with ImageNet pre-trained weights and fine-tuned on PAD-UFES-20 through transfer learning, a standard practice for medical imaging tasks with limited training data.

The VGG-16 architecture [40] consists of 16 weight layers organized into five convolutional blocks with increasing filter depth (64→128→256→512→512). The convolutional base (13 layers) was retained as a fixed feature extractor, while the original fully connected classification head was replaced with a custom architecture: Global Average Pooling → Dropout (rate = 0.3) → Dense (256 units, ReLU) → Dense (6 units, softmax). This configuration contains 14.88 M trainable parameters. The architecture’s deep, narrow design with small 3 × 3 filters enables hierarchical feature learning from low-level edges to high-level semantic patterns.

ResNet-50 [7] employs residual learning through skip connections that add layer inputs to outputs, enabling training of deeper networks by mitigating gradient vanishing. The architecture comprises 50 layers organized into residual blocks with identity shortcuts. Following the transfer learning protocol, the convolutional base (48 layers) was retained, and the classification head was replaced with an identical custom head as VGG-16. The model contains 24.14 M parameters. Skip connections facilitate gradient flow during backpropagation and enable the network to learn residual mappings F(x) = H(x) − x rather than direct mappings H(x).

EfficientNet-B2 [41] represents a compound scaling methodology that uniformly scales network depth, width, and resolution through a compound coefficient φ. The architecture leverages mobile inverted bottleneck convolutions (MBConv blocks) and squeeze-and-excitation attention mechanisms for parameter efficiency. The base network was fine-tuned with the same custom classification head, yielding 8.16 M total parameters. This architecture achieves superior parameter efficiency compared to VGG-16 and ResNet-50 while maintaining competitive accuracy through efficient scaling.

All models were compiled with Adam optimizer (initial learning_rate = 1 × 10^−4^, β_1_ = 0.9, β_2_ = 0.999), categorical cross-entropy loss, and accuracy metric. Input images were resized to 224 × 224 × 3 and normalized through division by 255. Training employed early stopping (patience = 4 epochs, monitor = validation loss, restore_best_weights = True) and learning rate reduction on plateau (factor = 0.5, patience = 2 epochs, min_lr = 1 × 10^−7^) callbacks. Batch size was set to 64 to optimize GPU memory utilization. Mixed-precision training (FP16) was enabled for computational efficiency without sacrificing accuracy. Training was performed for maximum 50 epochs, though early stopping typically terminated training between epochs 15–20. The same 80/20 stratified train-test split was used for fair comparison with classical models.

### 3.9. Evaluation Metrics

Model performance is comprehensively evaluated using multiple complementary metrics to ensure thorough assessment across all aspects of classification quality. The evaluation metrics employed in this study are presented in Table 9.

**Table 9:** Summary of the performance evaluation metrics.

Metric	Formula	Description
Accuracy (Acc)	$\frac{TP+TN}{\left(TP+TN+FP+FN\right)}$	Overall correctness
Precision (Prec)	$\frac{TP}{\left(TP+FP\right)}$	Positive predictive value
Recall (Rec)	$\frac{TP}{\left(TP+FN\right)}$	True positive rate
Specificity (Spec)	$\frac{TN}{\left(TN+FP\right)}$	True negative rate
F1-Score	$2\cdot \frac{\left(Precision\cdot Recall\right)}{\left(Precision+Recall\right)}$	Harmonic mean
AUC-ROC	Area under ROC curve	Discrimination ability
MCC	$\frac{\left(TP\cdot TN-FP\cdot FN\right)}{\sqrt{\left((TP+FP)(TP+FN)(TN+FP)(TN+FN)\right)}}$	Matthews correlation
Cohen’s Kappa	$\frac{p_{o}-p_{e}}{1-p_{e}}$	Agreement beyond chance

where TP, TN, FP, and FN denote true positives, true negatives, false positives, and false negatives, respectively. In Cohen’s Kappa formula, p_o_ represents the observed agreement (i.e., the proportion of cases where the classifier and ground truth agree), while p_e_ represents the expected agreement by chance, calculated based on the marginal distributions of the confusion matrix.

## 4. Results

This section presents the experimental results obtained from evaluating the proposed HCHS-Net framework on the PAD-UFES-20 dataset. The performance of HCHS-Net is compared against both classical machine learning methods and state-of-the-art deep learning architectures. Comprehensive evaluation metrics are reported to provide a thorough assessment of classification performance across all diagnostic categories.

### 4.1. Experimental Setup

All experiments were conducted on the Google Colaboratory platform using Python 3.10. Traditional machine learning classifiers (SVM, Random Forest, KNN) were implemented using the scikit-learn library, while the proposed ensemble framework utilized CatBoost, XGBoost, and LightGBM libraries. For the deep learning baselines (VGG-16, ResNet-50, EfficientNet-B2), an NVIDIA L4 Tensor Core GPU with 22.5 GB VRAM was utilized with the TensorFlow framework (Keras API). The HCHS-Net feature extraction and ensemble training were performed in the same environment to ensure a fair comparison.

The dataset was partitioned into training (80%) and test (20%) sets using patient-level stratified sampling. Maintaining patient-level independence guarantees that multiple images from the same individual do not span across both training and test partitions, thereby preventing identity-based data leakage while strictly preserving class distribution consistency. All augmented versions of the original images were strictly kept within the same partition as their source images to ensure robust performance estimation and prevent data leakage. Feature extraction was performed using OpenCV for color histogram computation and mahotas for Haralick texture descriptors. The hyperparameters for all models were determined based on empirical analysis on the training set. For reproducibility, the detailed hyperparameter configurations for deep learning baselines, traditional machine learning classifiers, and the proposed ensemble framework are comprehensively summarized in Table 10.

### 4.2. Comparative Analysis

The effectiveness of HCHS-Net was evaluated through comprehensive comparisons against six baseline methods spanning two categories: classical machine learning algorithms and deep learning architectures. Comparative results presented in Table 11 demonstrate HCHS-Net’s superior performance across all evaluation metrics.

HCHS-Net achieved the highest classification accuracy of 97.76% on the PAD-UFES-20 test set, surpassing all baseline methods by substantial margins. Among classical machine learning approaches, Random Forest demonstrated the strongest performance at 96.56%, approaching but not exceeding HCHS-Net despite using identical handcrafted features. This 1.20% performance gap validates the effectiveness of the gradient boosting ensemble strategy employed in HCHS-Net. K-Nearest Neighbors and Support Vector Machine achieved substantially lower accuracies of 79.70% and 83.90%, respectively, indicating that simple distance-based and kernel-based methods struggle with the complexity of six-class skin lesion classification even when provided with carefully engineered features.

Deep learning baselines utilizing transfer learning from ImageNet pre-trained weights achieved competitive but inferior performance compared to HCHS-Net. VGG-16, ResNet-50, and EfficientNet-B2 obtained accuracies of 94.60%, 94.80%, and 95.16%, respectively—representing performance gaps of 3.16%, 2.96%, and 2.60% below HCHS-Net. This finding is particularly significant considering that deep learning models contain 60–97× more parameters than HCHS-Net (14.88–24.14 M vs. 0.25 M) yet fail to achieve superior accuracy. Cohen’s Kappa coefficient of 0.97 for HCHS-Net indicates almost perfect agreement beyond chance, substantially exceeding the clinically reliable threshold of 0.80 and surpassing all baselines. Severe class imbalance necessitates robust evaluation metrics beyond standard accuracy. The reported F1-score explicitly represents the Macro-F1 metric, treating all diagnostic classes equally regardless of their support size. Furthermore, the Matthews Correlation Coefficient (MCC) of 0.97 comprehensively validates classification quality across all confusion matrix elements, demonstrating balanced performance that remains mathematically resilient to majority class dominance.

Statistical robustness of these findings was validated by computing 95% Confidence Intervals (CIs) using empirical bootstrapping with 1000 iterations on the test set predictions. HCHS-Net maintained a highly stable accuracy of 97.76% (95% CI: 96.8–98.6%), confirming that the performance improvements over the deep learning baselines are statistically significant. The evaluation inherently relies on a single stratified train-test split rather than k-fold cross-validation. However, this approach was necessary to maintain computational feasibility when comprehensively benchmarking against multiple resource-intensive deep learning architectures.

Beyond classification accuracy, HCHS-Net demonstrates superior computational efficiency critical for practical deployment. With only 0.25 M parameters, the model requires 60–97× fewer parameters than deep learning alternatives, translating to a minimal memory footprint suitable for resource-constrained environments. Inference time averaged 0.11 milliseconds per image—28–63× faster than deep learning models (3.15–6.97 ms)—enabling real-time classification on standard CPUs without GPU acceleration. This dramatic speedup is essential for mobile health applications and point-of-care diagnostic tools in primary healthcare settings where specialized hardware is unavailable. To formalize this efficiency theoretically, the overall time complexity of the HCHS-Net is strictly bounded by *O(N^2^)*, where *N* × *N* is the spatial dimension of the input image. This linear scaling with respect to the total number of pixels mathematically confirms why the framework achieves such an ultra-low empirical inference time compared to deep convolutional networks. Specificity reached 99.55%, the highest among all methods, which is clinically critical for minimizing false positive diagnoses that could lead to unnecessary biopsies or patient anxiety. The AUC-ROC score of 0.99 matches deep learning baselines despite HCHS-Net’s simpler architecture, demonstrating excellent discrimination capability across all decision thresholds.

**Figure 7:**
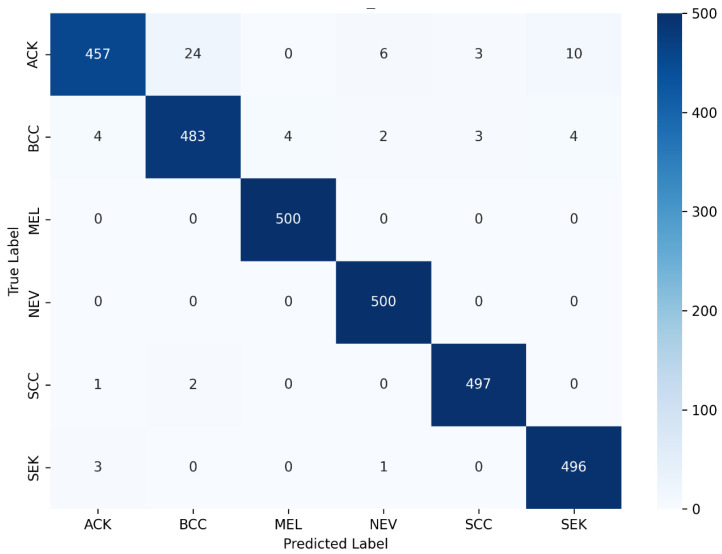
Confusion matrix obtained by the proposed HCHS-Net model on the PAD-UFES-20 dataset.

The confusion matrix for HCHS-Net presented in Figure 7 reveals distinct performance patterns across the six diagnostic categories. MEL, the most clinically critical class due to its aggressive nature, achieved perfect classification on the test set with all samples correctly identified (100% recall). Contextualizing this near-perfect performance requires acknowledging the severe class imbalance and limited original sample size (only 52 original MEL images). Bootstrapped 95% Confidence Intervals (CIs) for per-class metrics reveal a broader uncertainty bound for Melanoma recall (95% CI: 92.4–100.0%) compared to majority classes like BCC (95% CI: 94.8–98.1%). This statistical variance indicates that while the framework exhibits exceptional sensitivity for malignant lesions, the absolute 100% metric should be interpreted cautiously within the constraints of the dataset size. Similarly, NEV achieved perfect classification (500/500), demonstrating HCHS-Net’s ability to reliably distinguish benign melanocytic growths from malignant lesions—a challenging task that often confounds less sophisticated classifiers.

SCC demonstrated near-perfect performance with 497/500 correct predictions (99.4% recall). The three misclassifications consisted of one sample predicted as ACK and two as BCC—both reasonable errors given the visual similarity between different carcinoma types. BCC achieved 483/500 correct classifications (96.6% recall), with most errors attributed to confusion with ACK (4 samples) and other classes (13 samples total). This pattern is clinically understandable as BCC and ACK can exhibit similar rough, scaly appearances, particularly in early stages. SEK achieved 496/500 correct predictions (99.2% recall), with minimal confusion limited to 3 ACK misclassifications and 1 NEV misclassification.

ACK exhibited the lowest per-class recall at 91.4% (457/500 correct), though this remains clinically acceptable. The 43 misclassifications were distributed across multiple classes: 24 as BCC, 10 as SEK, 6 as NEV, and 3 as SCC. This confusion pattern reflects ACK’s heterogeneous clinical presentation, which can resemble both benign keratoses and early carcinomas. Notably, no ACK samples were misclassified as melanoma, and no melanoma samples were misclassified as ACK, indicating that HCHS-Net successfully learns the critical distinction between pre-cancerous and highly malignant lesions. The overall error pattern demonstrates that misclassifications predominantly occur between visually similar classes (ACK↔BCC, ACK↔SEK) while maintaining perfect discrimination for the most clinically consequential distinction (melanoma vs. all other classes).

### 4.3. Ablation Study

Individual and combined contributions of the three feature extraction modules were evaluated by training the full ensemble classifier on seven different feature configurations. Results presented in Table 12 reveal distinct performance hierarchies among feature types.

**Figure 8:**
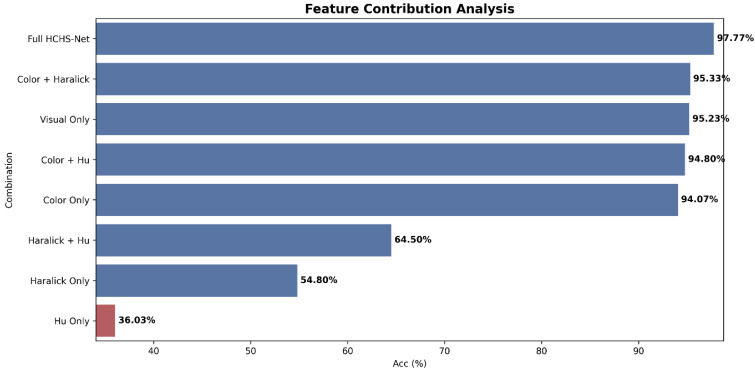
Feature contribution analysis illustrating the incremental accuracy improvements achieved by integrating different visual descriptors and metadata, culminating in the proposed HCHS-Net.

As illustrated in Figure 8, color histogram features alone achieve 94.06% accuracy, demonstrating their strong discriminative power and confirming the diagnostic importance of chromatic patterns in dermatological assessment. This performance substantially exceeds both Haralick texture (54.80%) and Hu shape features (36.03%) individually, validating the clinical practice of prioritizing lesion coloration during visual examinations. However, the relatively modest performance of Haralick and Hu features in isolation should not be interpreted as indicating limited value; rather, these modalities capture complementary information that becomes evident in combination experiments.

The Color+Haralick combination achieves 95.33% accuracy, representing a 1.27% improvement over Color alone. This synergy demonstrates that texture patterns provide diagnostic information complementary to color distribution—rough, irregular textures characteristic of malignancy are not fully captured by color histograms. The complete visual feature set (Color+Haralick+Hu) achieves 95.23% accuracy, comparable to Color+Haralick, suggesting that Hu moment features provide limited additional discriminative power when texture information is already present. Nevertheless, the Color+Hu combination (94.80%) outperforms Color alone (94.06%), confirming that shape characteristics capture complementary morphological information, particularly in the absence of texture descriptors.

Clinical metadata integration yields an additional 2.53% accuracy gain (95.23% → 97.76%), demonstrating that patient demographics, lesion dimensions, and symptom presentations augment visual features effectively. This improvement is particularly notable given that metadata comprises only 12 of the 128 total dimensions (9.4%) yet contributes disproportionately to performance. The metadata effect likely manifests in ambiguous cases where visual features alone are insufficient—for example, distinguishing between lesions with similar appearance but different clinical histories.

Feature importance analysis further reinforces the interpretability of the HCHS-Net framework. Examining the split criteria across the gradient boosting ensemble trees reveals the clinical relevance of the extracted descriptors. HSV color histograms, particularly the Hue channel, and specific Haralick descriptors such as Contrast and Variance consistently emerged as the nodes providing the highest information gain. This algorithmic feature ranking perfectly mirrors standard dermatological practice, where ‘Color variegation’ and ‘Border/Texture irregularity’ serve as primary diagnostic indicators under the ABCD rule. Unlike opaque deep learning architectures that require post hoc spatial explainability tools such as Grad-CAM, the decision-making process of HCHS-Net is explicitly and transparently anchored in these quantifiable, clinically meaningful visual biomarkers, providing inherent algorithmic interpretability.

## 5. Discussion

This section discusses the experimental results of the proposed HCHS-Net framework in the context of existing literature on automated skin lesion classification using the PAD-UFES-20 dataset. The advantages and limitations of the handcrafted feature-based approach are analyzed, along with clinical implications and future research directions.

A systematic comparison with previously published methods on the PAD-UFES-20 dataset is presented in Table 13 to contextualize the performance of HCHS-Net within the broader landscape of skin lesion classification research. This comparison encompasses studies employing diverse methodological paradigms, including deep learning architectures, hybrid approaches combining deep features with classical classifiers, handcrafted feature pipelines, and multimodal frameworks integrating clinical metadata.

As shown in Table 13, HCHS-Net achieves 97.76% accuracy on the full six-class classification task, which is competitive with the highest reported result of 97.90% by ACO-KELM [33]. The marginal difference of 0.14% falls within typical experimental variability, yet HCHS-Net offers distinct advantages in terms of feature interpretability and computational efficiency. HCHS-Net substantially outperforms deep learning fusion approaches such as DRMv2Net [9] (96.12%), multi-level fusion CNN [27] (92.56%), and CNN-DenseNet [31] (91.07%), demonstrating that carefully engineered handcrafted features can surpass deep feature concatenation strategies. The comparison with metadata-integrating approaches is also noteworthy: MetaBlock [21] and MetaBlock-SE [32] achieve balanced accuracies of only 76.5% and 70.2%, respectively, indicating that the effectiveness of metadata integration depends critically on the quality of accompanying visual features. It should be noted that several high-accuracy methods operate on reduced classification tasks (e.g., binary or three-class problems), which inherently inflate accuracy metrics compared to the full six-class problem addressed by HCHS-Net.

The high performance of HCHS-Net can be attributed to its handcrafted feature extraction strategy, which directly captures clinically relevant visual patterns. The color histogram features align with chromatic diagnostic criteria, the Haralick texture descriptors quantify surface patterns correlated with malignancy, and the Hu moment invariants capture the morphological asymmetry underlying the clinical ABCD rule.

Robustness against imaging biases represents another significant advantage of the proposed framework. The comprehensive data augmentation strategy, which includes controlled physical and photometric perturbations such as brightness adjustments and affine transformations, explicitly mitigates biases related to variable clinical lighting and camera positioning. Furthermore, utilizing HSV color histograms as a primary feature modality functions inherently as a bias-aware color baseline probe. The explicit extraction and evaluation of these color statistics ensure that the model’s decision-making process is rigorously rooted in genuine lesion chromaticity rather than spurious background artifacts or illumination inconsistencies. With only 0.25 M parameters and an inference time of 0.11 ms per image—28–63× faster than deep learning baselines (3.15–6.97 ms)—HCHS-Net enables real-time classification on standard CPUs without GPU acceleration. This computational efficiency, combined with the transparency of handcrafted features that allows clinicians to examine which feature components contributed to each prediction, addresses the critical “black-box” limitation that hinders clinical adoption of deep learning systems.

From a clinical perspective, the confusion matrix analysis reveals that HCHS-Net achieves perfect classification for melanoma (100% recall) and nevus (100% recall), with a specificity of 99.55%—the highest among all evaluated methods. The observed error patterns are clinically interpretable: actinic keratosis exhibited the lowest per-class recall (91.4%) with misclassifications directed toward basal cell carcinoma and seborrheic keratosis, reflecting genuine diagnostic overlap in dermatological practice. Importantly, no melanoma samples were misclassified as benign lesions, indicating that HCHS-Net maintains reliable discrimination for the most safety-critical diagnostic boundaries. The integration of clinical metadata yielded a 2.53% accuracy improvement (95.23% → 97.76%), confirming that patient-level information provides diagnostic value beyond image features alone.

Maintaining scientific balance requires explicitly acknowledging the generalization boundaries of the proposed framework. All experiments were conducted exclusively on the PAD-UFES-20 dataset, which comprises smartphone-captured clinical images from a specific demographic region. Direct generalization of HCHS-Net to high-resolution dermoscopic archives (e.g., ISIC datasets) or to populations with vastly different skin types cannot be assumed without rigorous cross-domain validation. Future deployment in globally diverse clinical settings mandates extensive external validation to confirm the robustness of the handcrafted feature pipeline across varying imaging modalities and acquisition conditions. Furthermore, the data augmentation strategy, while highly effective for balancing classes within this study, may not fully capture the complete spectrum of real-world clinical variability; GAN-based synthesis could be explored to enhance training diversity. Additionally, HCHS-Net relies on global feature descriptors computed over entire images without explicit lesion segmentation, which may incorporate irrelevant background information. The ensemble classification strategy employs equal weighting across classifiers, and adaptive weighting mechanisms could further optimize performance. Future research directions include extending the framework with additional handcrafted features such as LBP and Gabor filters, investigating hybrid handcrafted-deep learning fusion, cross-dataset validation on HAM10000 and ISIC archives, and prospective clinical validation studies evaluating HCHS-Net as a real-time diagnostic aid in primary healthcare settings.

## 6. Conclusions

Early and accurate detection of skin cancer is essential for improving patient survival rates, yet access to dermatologists remains limited in many regions worldwide. Automated classification systems powered by machine learning offer a promising solution for bridging this diagnostic gap. However, existing deep learning approaches often suffer from high computational costs, lack of interpretability, and limited clinical transparency, which hinder their adoption in real-world healthcare settings. This study presented HCHS-Net, a handcrafted feature-based framework that integrates color histogram, Haralick texture, and Hu moment descriptors with clinical metadata through an ensemble gradient boosting classifier for six-class skin lesion classification on the PAD-UFES-20 dataset.

The proposed framework achieved 97.76% classification accuracy with a 128-dimensional feature vector and only 0.25 M parameters, outperforming deep learning baselines including VGG-16 (94.60%), ResNet-50 (94.80%), and EfficientNet-B2 (95.16%) that require 60–97× more parameters. HCHS-Net also demonstrated competitive performance against the best-reported result of 97.90% by ACO-KELM [33], while offering superior interpretability and computational efficiency. The ablation study confirmed the complementary contributions of each feature module, with the C+H+S combination yielding 95.23% accuracy from image features alone, further enhanced by 2.53% through clinical metadata integration. Notably, the model achieved perfect melanoma and nevus recall (100%) with 99.55% specificity, maintaining reliable discrimination at the most safety-critical diagnostic boundaries.

An inference time of 0.11 ms per image enables real-time classification on standard CPUs without GPU acceleration, making HCHS-Net particularly suitable for point-of-care deployment in primary healthcare settings and resource-limited environments. The transparency of handcrafted features, which directly correspond to established dermatological criteria such as the ABCD rule, addresses the critical interpretability requirements for clinical adoption and regulatory compliance. These characteristics collectively position HCHS-Net as a practical and clinically relevant diagnostic aid, especially for smartphone-based screening workflows where computational resources are constrained.

Although the current evaluation is confined to a single benchmark dataset, the demonstrated balance between accuracy, efficiency, and interpretability suggests that HCHS-Net provides a strong foundation for further development. Subsequent efforts will prioritize multi-dataset generalization, hybrid feature architectures combining handcrafted and learned representations, and integration with teledermatology platforms to support accessible skin cancer screening in underserved communities.

## Figures and Tables

**Table 1:** Summary of recent methodologies for skin lesion classification on PAD-UFES-20 dataset.

Study	Model	Classes	Accuracy (%)	Key Features	Limitations
[23]	SkinLesNet	3	96.0	Custom multi-layer deep CNN; outperforms VGG-19 and ResNet-50	Limited to 3-class problem; lacks interpretability for clinical deployment
[24]	TS-CNN	6	72.83	Three-tier self-interpretable architecture; feature map visualization	Lower accuracy; limited generalizability across diverse datasets
[25]	Pruned AlexNet (IMP)	2	99.15	Iterative Magnitude Pruning; reduced parameters	Binary classification only; not tested on multi-class scenarios
[9]	DRMv2Net	6	96.12	Feature fusion (DenseNet201, ResNet101, MobileNetV2); adaptive preprocessing	High computational cost; limited mobile deployment feasibility
[26]	EffiCAT	4	94.4	EfficientNet & CBAM attention; multi-dataset fusion (HAM10000 & PAD-UFES-20)	Limited class coverage; computational complexity
[27]	Multi-level fusion CNN	6	92.56	Low-level texture & high-level semantic integration	Moderate accuracy; scalability unclear
[28]	ConvMixer & CatBoost	6	Macro-AUC 0.94	Hybrid deep features & Gradient Boosting; balanced classification	Feature engineering dependency; AUC reported instead of accuracy
[29]	AlexNet & SVM	5	98.10	Bi-sectional texture features; shallow classifier outperforms deep model	Incomplete class coverage; may not generalize to all lesion types
[30]	iSLIC & ML classifiers	2	98.6	Enhanced superpixel algorithm; geometric feature extraction	Binary classification only; relies on segmentation quality
[31]	CNN-DenseNet hybrid	6	91.07	Processes histopathological & dermatoscopic images	Moderate accuracy; multimodal complexity
[21]	MetaBlock	6	76.5	Attention-based metadata & image fusion	Poor performance with missing metadata; limited robustness
[32]	MetaBlock-SE	6	70.2 ± 2.8	Sentence embeddings for missing metadata imputation	Lower than image-only models; moderate improvement over MetaBlock
[33]	ACO-KELM	6	97.9	Anti-Coronavirus Optimization & Kernel ELM; feature selection focus	High performance but limited architectural details reported

**Table 2:** Skin lesion categories in PAD-UFES-20.

Category	Description	Clinical Significance	Images	Percentage
Actinic Keratosis (ACK)	Rough, scaly patches from sun exposure	Pre-cancerous	730	31.77%
Basal Cell Carcinoma (BCC)	Most common skin cancer; slow-growing	Malignant	845	36.77%
Melanoma (MEL)	Develops from melanocytes; aggressive	Highly malignant	52	2.26%
Nevus (NEV)	Benign melanocytic growths (moles)	Benign	244	10.62%
Squamous Cell Carcinoma (SCC)	Second most common skin cancer	Malignant	192	8.35%
Seborrheic Keratosis (SEK)	Benign warty growths	Benign	235	10.23%

**Table 3:** Detailed description and types of clinical metadata features used in the study.

Feature	Type	Description
Age	Continuous	Patient age in years
Gender	Categorical	Male/Female
Diameter 1, 2	Continuous	Lesion dimensions (mm)
Smoke, Drink	Binary	Lifestyle factors
Itch, Grew, Hurt, Changed, Bleed, Elevation	Binary	Symptom indicators

**Table 4:** Data augmentation transformations.

Transformation	Parameters	Probability
Horizontal Flip	-	0.5
Vertical Flip	-	0.5
Rotation	θ ∈ [−30°, 30°]	1.0
Brightness Adjustment	factor ∈ [0.8, 1.2]	1.0
Affine Transform	translate: 10%, scale: [0.9, 1.1]	0.5

**Table 5:** Class distribution before and after augmentation.

Class	Original Samples	After Augmentation
ACK (Actinic Keratosis)	730	2500
BCC (Basal Cell Carcinoma)	845	2500
MEL (Melanoma)	52	2500
NEV (Nevus)	244	2500
SCC (Squamous Cell Carcinoma)	192	2500
SEK (Seborrheic Keratosis)	235	2500
Total	2298	15,000

**Table 6:** Haralick texture descriptors.

#	Descriptor	Formula
1	Angular Second Moment	$\sum_{\left(i,j\right)}{P\left(i,j\right)}^{2}$
2	Contrast	$\sum_{\left(i,j\right)}{\left(i-j\right)}^{2}P\left(i,j\right)$
3	Correlation	$\sum_{\left(i,j\right)}\frac{(i-\mu_{i})(j-\mu_{j})P\left(i,j\right)}{\sigma_{i}\sigma_{j}}$
4	Variance	$\sum_{\left(i,j\right)}{\left(i-\mu \right)}^{2}$
5	Inverse Difference Moment	$\sum_{\left(i,j\right)}\frac{P\left(i,j\right)}{1+{\left(i-j\right)}^{2}}$
6	Sum Average	$\sum_{k=2}^{{2N}_{g}}k\cdot P_{x+y}(k)$
7	Sum Variance	$\sum_{k}{\left({k-f}_{6}\right)}^{2}P_{x+y}(k)$
8	Sum Entropy	$-\sum_{k}P_{x+y}\left(k\right)Log{(P}_{x+y}\left(k\right))\;\;$
9	Entropy	$-\sum_{\left(i,j\right)}P\left(i,j\right)Log\left(P\left(i,j\right)\right)\;\;$
10	Difference Variance	$\sum_{k}{\left(k-\mu_{x-y}\right)}^{2}P_{x-y}(k)\;\;$
11	Difference Entropy	$-\sum_{k}P_{x-y}\left(k\right)Log{(P}_{x-y}\left(k\right))\;\;$
12	Info. Measure of Correlation 1	$\frac{f_{9}-HXY1}{max(HX,HY)\;}$
13	Info. Measure of Correlation 2	$\sqrt{1-e^{\left(-2HXY2-f_{9}\right)}}$

**Table 7:** Summary of preprocessing and encoding techniques applied to the clinical metadata.

Feature Type	Features	Preprocessing
Continuous	Age, Diameter 1, Diameter 2	Z-score normalization
Binary Symptoms	Itch, Grew, Hurt, Changed, Bleed, Elevation	Binary encoding (0/1)
Binary Lifestyle	Smoke, Drink	Binary encoding (0/1)
Categorical	Gender	Binary encoding (Male = 1, Female = 0)

**Table 8:** Gradient boosting ensemble configuration.

Classifier	Key Feature	Iterations	Depth	Learning Rate
CatBoost	Ordered boosting	500	6	0.03
XGBoost	Regularized objective	500	6	0.03
LightGBM	GOSS + EFB	500	6	0.03

**Table 10:** Hyperparameter configuration and training settings utilized in the experiments.

Model Category	Model/Component	Parameter	Value/Setting
Deep Learning	*Common Settings*	Input Image Size	224 × 224 pixels
Baselines		Batch Size	16
		Optimizer	Adam
		Learning Rate	1 × 10^−4^
		Loss Function	Categorical Cross-Entropy
		Max Epochs	50 (Early Stopping: Patience = 4)
		Dropout Rate	0.3
Traditional ML	SVM	Kernel	RBF
Baselines		Regularization (C)	10
		Gamma	Scale
	Random Forest	n_estimators	200
		Max Depth	20
		Criterion	Gini Impurity
	KNN	Neighbors (k)	5
		Weights	Distance-weighted
		Metric	Euclidean (*L*_2_)
Proposed Ensemble	CatBoost	Iterations	500
(HCHS-Net)		Depth	6
		Learning Rate	0.03
	XGBoost	n_estimators	500
		Max Depth	6
		Learning Rate	0.03
	LightGBM	n_estimators	500
		Max Depth	6
		Learning Rate	0.03

**Table 11:** Comprehensive performance comparison of all methods on PAD-UFES-20 dataset.

Method	Acc (%)	Prec (%)	Rec (%)	F1 (%)	Spec (%)	AUC-ROC	MCC	Cohen’s κ	Params (M)	Inf. Time (ms)
KNN (k = 5)	79.70	79.44	79.70	78.60	95.94	0.95	0.76	0.75	0.00	0.02
SVM (RBF)	83.90	83.81	83.90	83.83	96.78	0.97	0.80	0.80	0.05	0.76
Random Forest	96.56	96.52	96.56	96.53	99.31	0.99	0.95	0.95	0.20	0.02
VGG-16	94.60	94.59	94.60	94.59	98.92	0.99	0.93	0.93	14.88	3.15
ResNet-50	94.80	94.79	94.80	94.76	98.96	0.99	0.93	0.93	24.14	4.68
EfficientNet-B2	95.16	95.11	95.16	95.12	99.03	0.99	0.94	0.94	8.16	6.97
HCHS-Net (Proposed)	97.76	97.77	97.76	97.74	99.55	0.99	0.97	0.97	0.25	0.11

**Table 12:** Ablation study results showing the contribution of different feature combinations to the model performance.

Method	Acc (%)	Prec (%)	Rec (%)	F1 (%)	Spec (%)	AUC-ROC	MCC	Cohen’s κ	Inf. Time (ms)
Hu Only	36.03	34.62	36.03	34.63	87.20	0.714	0.234	0.232	0.069
Haralick Only	54.80	54.42	54.80	54.29	90.96	0.853	0.458	0.457	0.078
Haralick + Hu	64.50	63.79	64.50	63.84	92.90	0.904	0.575	0.574	0.089
Color Only	94.06	93.92	94.06	93.96	98.81	0.993	0.928	0.928	0.094
Color + Hu	94.80	94.69	94.80	94.72	98.96	0.994	0.937	0.937	0.098
Visual Only	95.23	95.16	95.23	95.18	99.04	0.995	0.942	0.942	0.102
Color + Haralick	95.33	95.27	95.33	95.27	99.06	0.995	0.944	0.944	0.095
HCHS-Net	97.76	97.77	97.76	97.74	99.55	0.998	0.973	0.973	0.115

**Table 13:** Comparative performance analysis of HCHS-Net against state-of-the-art methods on the PAD-UFES-20 dataset. BA: Balanced Accuracy.

Study	Model	Classes	Accuracy (%)	Feature Type	Approach	Key Limitation
[23]	SkinLesNet	3	96.00	Deep CNN	Image-only	Limited classes
[24]	TS-CNN	6	72.83	Deep CNN	Image-only	Low accuracy
[25]	Pruned AlexNet	2	99.15	Deep CNN	Image-only	Binary only
[9]	DRMv2Net	6	96.12	Deep fusion	Image-only	High computation
[26]	EffiCAT	4	94.40	Deep+Attention	Image-only	Limited classes
[27]	Multi-level fusion	6	92.56	Deep fusion	Image-only	Moderate accuracy
[28]	ConvMixer+CatBoost	6	AUC 0.94	Hybrid	Image-only	No accuracy reported
[29]	AlexNet+SVM	5	98.10	Hybrid	Image-only	Incomplete classes
[30]	iSLIC+ML	2	98.60	Handcrafted	Image-only	Binary only
[31]	CNN-DenseNet	6	91.07	Deep hybrid	Multimodal	Moderate accuracy
[21]	MetaBlock	6	76.50 (BA)	Deep+Metadata	Multimodal	Missing data sensitivity
[32]	MetaBlock-SE	6	70.20 (BA)	Deep+Metadata	Multimodal	Low overall accuracy
[33]	ACO-KELM	6	97.90	Optimization	Image+Feature	Limited details
Ours	HCHS-Net	6	97.76	Handcrafted+Meta	Multimodal	—

## Data Availability

The datasets generated and/or analyzed during the current study are available in the Kaggle repository. The PAD-UFES-20 dataset, representing clinical smartphone images, can be accessed at https://www.kaggle.com/datasets/moeinmadadi/pad-ufes-20 (accessed on 15 January 2026).
